# NS1: A Key Protein in the “Game” Between Influenza A Virus and Host in Innate Immunity

**DOI:** 10.3389/fcimb.2021.670177

**Published:** 2021-07-13

**Authors:** Zhu-xing Ji, Xiao-quan Wang, Xiu-fan Liu

**Affiliations:** ^1^ Animal Infectious Disease Laboratory, School of Veterinary Medicine, Yangzhou University, Yangzhou, China; ^2^ Jiangsu Co-innovation Center for Prevention and Control of Important Animal Infectious Diseases and Zoonosis, Yangzhou University, Yangzhou, China; ^3^ Jiangsu Key Laboratory of Zoonosis, Yangzhou University, Yangzhou, China; ^4^ Key Laboratory of Prevention and Control of Biological Hazard Factors (Animal Origin) for Agri-food Safety and Quality, Ministry of Agriculture of China (26116120), Yangzhou University, Yangzhou, China

**Keywords:** influenza, NS1, innate immunity, protein-protein interaction, post-translational modification

## Abstract

Since the influenza pandemic occurred in 1918, people have recognized the perniciousness of this virus. It can cause mild to severe infections in animals and humans worldwide, with extremely high morbidity and mortality. Since the first day of human discovery of it, the “game” between the influenza virus and the host has never stopped. NS1 protein is the key protein of the influenza virus against host innate immunity. The interaction between viruses and organisms is a complex and dynamic process, in which they restrict each other, but retain their own advantages. In this review, we start by introducing the structure and biological characteristics of NS1, and then investigate the factors that affect pathogenicity of influenza which determined by NS1. In order to uncover the importance of NS1, we analyze the interaction of NS1 protein with interferon system in innate immunity and the molecular mechanism of host antagonism to NS1 protein, highlight the unique biological function of NS1 protein in cell cycle.

## Introduction

Influenza is a global respiratory infectious disease. Every year, about 290,000-650,000 people die from respiratory diseases caused by influenza (Feng et al., 2012). It is important to be deeply aware of the global public health threat posed by influenza. In the history of mankind, there have been a total of four influenza pandemics, namely Spanish Influenza in 1918, Asian Influenza in 1957, Hong Kong Influenza in 1968, and 2009 pandemic influenza (Jennings, 2007). In recent years, although there has not been a pandemic of influenza, there is a continuing epidemic trend. From 2019 to 2020, the World Health Organization’s GISRS laboratory tested more than 174604 samples. A total of 44,847 people tested positive for influenza virus, of which 27,946 (62.3%) were influenza A virus (World Health Organization). It is easy to observe from these data that influenza virus is indeed a great threat to human public health. The persistence and widespread presence of influenza viruses is due to multiple factors.

Influenza A virus (IAV) is highly polymorphous, exhibiting either spherical or filamentous. Influenza virus is an enveloped virus, whose envelope comes from the host cell membrane with lipid bilayer structure. IAV contain 8 single-stranded negative-strand RNA fragments, which are PB2, PB1, PA, HA, NP, NA, M, and NS, respectively. RNA fragment can be capsized to form ribonucleoprotein (RNP) wrapped by M1 protein. Each of the eight RNPs contains a fragment of the influenza virus genome, which is the smallest functional unit of genome transcription and replication (Long et al., 2019).

The vRNP complex utilizes the nuclear localization signal of NP proteins to enter the nucleus (O’Neill et al., 1995). Subsequently, viral RNA (vRNA) is transcribed into mRNA, a process that requires robbing the 5’ end of the cap structure from the host cell mRNA precursor transcript (Engelhardt et al., 2005). After completing the “cap-snatch” step, PB1 catalyzes the synthesis and extension of mRNA (Braam et al., 1983). During the termination phase, the influenza virus polyadenylates mRNA in a unique way, its poly(A) tails added by viral polymerase (Vreede et al., 2010), which diffident from mechanism of host cells: the polyadenylation signal is recognized by the host CPSF30 and cleaved, and the poly A polymerase (PAP) add Poly(A) tail (Robertson et al., 1981). During viral replication, the mRNAs of the eight viral gene segments are not uniformly distributed in a time-dependent manner, with mRNA transcription and protein synthesis of NS1 occurring early after viral infection (Shapiro et al., 1987), which may contribute to the promotion of polymerase complex activity due to NS1 (Mahmoudian et al., 2009). And NS1 can also bind to eIF4G I, thus promoting the translation of mRNA (Marión et al., 1997). In addition to this, NS1 can help the influenza virus gain an early advantage in the “game” by suppressing host gene expression and other ways to counteract the innate immune response.

IAV is constantly mutating during the evolutionary process, and 18 different HA subtypes and 11 different NA subtypes have been identified, but different subtypes of influenza viruses are also constantly undergoing genetic mutations and genetic recombination to produce new viruses, and some of the more pathogenic strains pose a great threat to the health of humans and animals. Although influenza A viruses have evolved to various subtypes, their ultimate ability to infect the host and cause disease in the organism is the result of the interaction between the virus and the host. In this process, influenza viruses need to break through the first line of defense against pathogenic invasion: innate immunity, which can limit the replication of the virus in the early stages of infection through the interferon (IFN) system (Goodman et al., 2010). Non-structural protein 1 (NS1) is the key protein in this “game”, its main role is to counteract the host’s natural immune response and inhibit interferon production.

Here, we focus on the NS1 protein and illustrate its effects on IAV virulence and pathogenicity, as well as the molecular mechanisms by which the NS1 protein antagonizes host innate immunity and interferon pathways, providing potential targets and molecular mechanisms for the development of new antiviral drugs.

## Influenza A Virus and Innate Immunity

Unlike most other RNA viruses, IAV replicates in the nucleus instead of in the cytoplasm. Nuclear replication requires transport through the cell membrane, input of vRNA containing template of viral RNPs, export and maturation of viral proteins and viral RNA (Talon et al., 2000).

This biological characteristic makes the interaction between influenza virus and host unique and complex. IAV requires the host cell for complete replication processes such as transcription, transport and protein expression to facilitate RNA replication of newly assembled viruses, packaging and budding of sub-generational viruses. The innate immune system is the organism’s first line of defense against microbial invasion. It inhibits the replication and spread of the initial virus. In addition, it protects surrounding uninfected cells and stimulates macrophages and dendritic cells and other immune cells (Banchereau and Steinman, 1998; Wolff and Ludwig, 2009). To fight against viral invasion, the body’s innate immune system has a molecular mechanism to fight microbial invasion —— the interferon system.

The 5’ end and 3’ end of influenza virus RNA contain partial complementary sequences, which can form a short 5’ triphosphate double stranded RNA (5’ ppp-dsRNA) structure (Pflug et al., 2014). It can be recognized by specific pattern recognition receptors (PRs) as pathogen-associated molecular patterns (PAMPs), which can trigger downstream transcriptional activators, such as interferon regulatory factors 3 and 7 (IRF3 and IRF7), NF-κB and activator protein 1(AP-1), ultimately contributing to type I IFN production (**Figure 2**). When IAVs are recognized as PAMPs, Toll-like receptors (TLRs) and Retinoic acid-inducible gene I-like receptors (RLRs) are the primary pathways for inducing type I IFN responses (Takeuchi and Akira, 2010).

TLRs located on the cell surface or in the nucleus (Akira et al., 2006). TLR3, TLR7 and TLR8 are expressed in a variety of cells within the respiratory mucosa. TLR3 recognizes dsRNA and is expressed in respiratory epithelial cells, alveolar macrophages and dendritic cells (Guillot et al., 2005), whose main function is to induce the expression of IFNA and proinflammatory cytokines to deal with IAV infection (Le Goffic et al., 2007). The recognition of viral RNA by TLRs activates the IFN signal pathway with the help of intracellular receptors (Alexopoulou et al., 2001). When activated by viral dsRNA, TLR7 interacts with Myeloid Differentiation Factor 88(MyD88) in plasma cell-like dendritic cells (DCs), leading to activation of IRF7 and NF-κB, which control high levels of IFN-β and IFN-α transcription (Lund et al., 2004). Differing from TLR7, TLR3 activation recruits TRIF through its own TIR domain (Yamamoto et al., 2003), which in turn binds TRAF3/6 and then activates the protein kinases TBK1/IKK-ϵ and IKK/IKK-β (Fitzgerald et al., 2003; Häcker et al., 2006; Chau et al., 2008). Ultimately, the transcription factors IRF3/7 and NF-κB translocate to the nucleus and cause activation, inducing type I IFN to protect against viral invasion (Sharma et al., 2003; Seth et al., 2005).

RLRs expressed in the cytoplasm can recognize viral RNA (Tripathi et al., 2015). It has been proved that IFN can still be produced in TLR3-deficient cells (Edelmann et al., 2004), because of the presence of viral RNA recognition sensors, namely RIG-I, MDA5 and LGP, in the cytoplasm, which are also called RLRs (Yoneyama et al., 2004). RIG-I in the cytoplasm is the main PRR, for the detection of influenza virus in epithelial cells and the main target for influenza virus infection *in vivo*. In non-induced conditions, RIG-I is inactive in the cytoplasm. However, when RIG-I recognizes to the 5’ppp dsRNA, forming a dimer formation, the CARD domain will be exposed (Takahasi et al., 2008; Cui et al., 2008). Subsequently, TRIM25 binds to the Lysine residue at position 172 of the CARD domain (Gack et al., 2008), which could allow RIG-I transfer to mitochondria for binding to MAVS (Gack et al., 2007) (**Figure 2**). MAVS activates the protein kinases TBK-1 and IKK-ϵ by recruiting TRAF3, which leads to phosphorylation of IRF3/7. Activated IRF3/7 translocates to the nucleus, thereby inducing type I IFN production (Hou et al., 2011).

Next, interferon binds with IFNAR to form a complex to activate the JAK-STAT pathway and downstream signals, and induce ISGs production (Marcello et al., 2006; Bender et al., 2015). Members of JAK-STAT protein family play a key role in signaling pathway.When IFN binds to its receptor, JAK1 and TYK2 are activated by phosphorylation. STAT1 in its non-activated state is phosphorylated by JAK1 after binding to the receptor through its SH2 structural domain, thus forming a dimer that enters the nucleus (Heim et al., 1995), which drives the expression of hundreds of ISGs (Shuai et al., 1992; Greenlund et al., 1995), such as Mxs, IFITM, PKR and OAS- RnaseL, etc., to restrict IAV replication (Villalón-Letelier et al., 2017).

In general, when influenza A virus invades the body, IFN-α/β secreted by the host cell binds to the commonly expressed interferon alpha/beta receptor, inducing the JAK-STAT signaling pathway and producing hundreds of interferon-stimulated genes. This induces the cell to enter an “antiviral state”, thereby limiting further virus replication and propagation (Ivashkiv and Donlin, 2014). Notably, as the “war” escalates, influenza also has a unique strategy to counteract the limitations of the interferon system, namely the NS1 protein. It can restrict interferon signaling at multiple levels, or antagonize ISGs, to improve the survival or replication efficiency of the influenza virus. We will elaborate on this in the following sections.

## NS1 Protein and Pathogenicity

### Characterization of NS1 Protein

Most viruses have evolved unique ways to overcome the immune system and maintain their survival. Among them, RNA viruses produce viral proteins that facilitate their own replication and effectively evade the antiviral response to interferon as needed to improve their survival rate (Randall and Goodbourn, 2008). NS1 is an important immunomodulatory factor whose primary role is to inhibit interferon production and counteract the activity of interferon-inducible proteins that limit influenza virus replication (Price et al., 2000).

The influenza A virus NS1 protein is between 230 and 237 amino acids in length and has a molecular weight of approximately 26 kDa (Hale et al., 2008). The NS1 protein is composed of two different domains. The first 73 amino acids constitute double-stranded RNA binding domain (RBD), while the latter 85-207 amino acids constitute the effector domain (ED). The two are connected by a flexible linker region (LR). The last 30 residues form a C-terminal disordered tail(CTT).

NS1 forms a homodimer, by dimerizing RBD and ED with the corresponding same domains to achieve binding to another NS1 monomer (Nemeroff et al., 1995). RBD is a six-helix symmetrical arrangement, and each monomer has three α-helices (Cheng et al., 2009). The dimerization of RBD requires the winding of double-stranded RNA (Nogales et al., 2018), where the key sites are the key amino acids R38 and K41 that interact with dsRNA (Talon et al., 2000). The interaction between EDs through the highly conserved W187 leads to weak dimerization of the NS1 protein, which contributes to the cooperative binding of the full-length NS1 protein and dsRNA (Hale et al., 2008).

Besides, NS1 contains a nuclear localization signal (NLS) in the RBD. The amino acid length of NLS is between 35-41 residues, which can bind to importin-α and be beneficial to transport through the nuclear membrane. H3N2 and H2N2 influenza viruses have a second NLS at the CTT, which can also localize the NS1 protein in the nucleus (Melén et al., 2007). Besides, the influenza virus NS1 contains a nuclear export signal (NES) lurking at the end of the ED domain at residues 138-147 (Li et al., 1998). Before 1940, HIN1 IAV contained NS1 protein with a length of 230 amino acids. Around 1940, mutation in the NS1 sequence of influenza virus extended the tail by 7 amino acids. This extended NS1 sequence added new localization signals and potential cell protein binding sites for itself (Melén et al., 2007), and its characteristics were consistent with those of the NS1 of influenza viruses of H3N2 and H2N2 subtypes.

The LR region and disordered C-terminal amino acid sequence of NS1 protein showed high variation. The LR region affects the relative spatial position of the two domains of NS1 protein, which is an important factor affecting the pathogenicity (Bornholdt and Prasad, 2008). More than 90% of H5N1 influenza virus lack 5 amino acids (García-Sastre et al., 1998; Pichlmair et al., 2006; Fernandez-Sesma et al., 2006; Wang et al., 2017; Hu and Shu, 2018) in their link regions, resulting in increased virulence (Lipatov et al., 2005; Long et al., 2008). In recent years, however, the strains with 5 amino acids added to the 80-84 positions of the NS1 protein has become prevalent in H5N1 subtype AIVs isolated from mammals. Our laboratory findings suggest that this H5N1 subtype of influenza virus can strongly induce an innate immune response, leading to a strong cytokine storm and enhanced pathogenicity in mammals (Chen et al., 2020). In terms of the C-terminal amino acid sequence, the GSEI and EPEV motifs of the NS1 protein were involved in viral virulence and aided the A/Swine/Shanghai/1/2005 virus in crossing the species barrier from swine to mice (Wang et al., 2012).

ED mainly interacts with cellular proteins to regulate their function: ED is important for RBD localization, NS1 nuclear output, inactivation of Cleavage and polyadenylation specificity factor 30 kDa (CPSF30) (Hale et al., 2008). These two domains of NS1 protein are quite conserved. If there is a mutation in its amino acid site, it may have a strong effect on its ability to act and the virulence of the strain.

### Genetic Mutation

Upon infection of cells by IAV, the process of replication, transcription, and synthesis of viral proteins is heavily dependent on cellular mechanisms in which certain factors in the host cell are able to restrict viral replication, and thus the proteins of influenza viruses have evolved to produce adaptive mutations to antagonize the restrictions of natural host immunity or even to help the virus break through the interspecies barrier. NS1, an influenza virus an important protein for suppression and evasion of innate immunity, changes in its amino acid sites have had an impact on biological properties, with some site-specific mutation significantly enhancing the pathogenicity of influenza A viruses.

The NS1 protein of the highly pathogenic H5N1 influenza virus isolated in the 1997 pandemic differs from other influenza viruses, and the pathogenicity of recombinant viruses containing this subtype of NS1 protein is significantly enhanced (Seo et al., 2002). It was later found that the amino acid substitution D92Q largely affected the pathogenicity of the virus in pigs and mice (Lipatov et al., 2005).

The 42nd amino acid located in the RBD of the NS1 protein has an important role in regulating the pathogenicity of influenza viruses, antagonizing interferon induction in host cells. The P42S mutation in H5N1 avian influenza virus allowed the NS1 protein to block the activation of the double-stranded RNA-mediated NF-κB pathway and the IRF-3 pathway (Jiao et al., 2008). Among H1N1 subtype influenza viruses, when amino acid 42 is S can also regulate the host IFN response and promote viral replication by blocking the activation of IRF3 (Cheng et al., 2018).

In addition to this, many mutations occurring on the NS1 protein can improve its antagonistic ability against IFNs and ISGs, and we summarize the more important ones in the following table (**Table 1**).

**Table 1:** Effects of changes of amino acid sites in NS1 on biological characteristics.

IAV	NS1 Amino Acid Residue	Biological effects	Impact on infection	Experiment method	Reference
A/chicken/China/B1-6/2006(H9N2)	X35R X46R	Losing its RNA silencing suppression activity.	Unreported	*In vitro*	(Jing et al., 2015)
A/swine/Shanghai/3/2014(H1N1)	S42	Regulating the host IFN response by blocking the activation of IRF3	Facilitating virus replication	*In vitro*	(Cheng et al., 2018)
A/Duck/Guangxi/12/03(H5N1)	P42S	Preventing the dsRNA-mediated activation of the NF-κB pathway and the IRF-3 pathway	Attenuating virus replication	*In vitro*	(Jiao et al., 2008)
A/chicken/Hunan/1/2009 (H5N1)	K55E/K66E/C133F	Restoring the ability to bind CPSF30 and reduced interferon response activity.	Increasing virus titer and replication efficiency	*In vitro*	(Li et al., 2018)
Recombined A/Puerto Rico/8/34 (H1N1)	I64T	Decreasing general inhibition of host protein synthesis by decreasing its interaction with CPSF30	Exhibiting an attenuated phenotype	*In vivo*	(DeDiego et al., 2016)
A/Duck/Hubei/2004/L-1(H5N1)	Y84F	Abolishing NS1-mediated downregulation of IFN-inducible STAT phosphorylation, and surface IFNAR1 expression	Reducing lung viral titers and increased lung ISG expression	*In vivo*	(Wang et al., 2017)
A/Hong Kong/156/97(H5N1)	D92E	Activating phosphorylation of NS1	Unreported	*In silico*	(Li and Wang, 2007)
A/quail/Hong Kong/G1/97(H9N2)	L103F/I106M/P114/G125D/N139D	Restoring CPSF30 binding capacity and inhibiting host gene expression	Unreported	*In vitro*	(Rodriguez et al., 2018)
A/Hong Kong/156/1997(H5N1)	F103L M106I	Increasing the ability of IFN antagonism, altering RIG-I and CPSF30 host factor binding ability	Increasing viral replication in mouse lungs	*In vivo*	(Dankar et al., 2013)
A/Hong Kong/1/68(H3N2)	F103L M106I		Increasing tropism and virulence in mouse lungs	*In vivo*	(Dankar et al., 2011)
Recombined A/Shanghai/1/2013 (H7N9)	I106M	Restoring the ability of CPSF30 binding and that of block host gene expression	Showing enhanced replication and virulence	*In vivo*	(Ayllon et al., 2014)
Recombined A/Puerto Rico/8/34 (H1N1)	A171Y	Decreasing expression of IFN and ISGs	Unknown	*In vitro*	(Plant et al., 2017)
A/Udorn/72(H1N1)	G184R	An unknown mechanism independent of overactivation of the host IFN response or to enhanced sensitivity of these viruses to the antiviral effect of PKR	Remarkable attenuation of virulence	*In vivo*	(Steidle et al., 2010)
A/canine/NY/dog23/2009(H3N8)	K186E	Introducing both the NS1-CPSF30 interaction and ISGs gene expression inhibition	Unreported	*In vitro*	(Nogales et al., 2017) (Chauché et al., 2018)
Recombined A/Puerto Rico/8/34 (H1N1)	D189N	Impairing the ability of the NS1 protein to inhibit general gene expression	Attenuating virulence	*In vitro* *In vivo*	(Nogales et al., 2017)
	V194I	Demonstrating a temperature-sensitive phenotype	Attenuating virulence more than D189N		

### Post-Translational Modification

**Figure 1:**
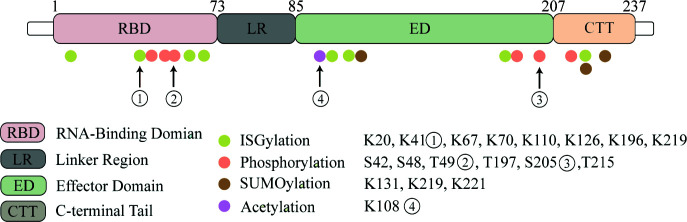
NS1 protein undergoes post-translational modifications. Four kinds of post-translational modifications can be made to NS1, namely: ISGylation, phosphorylation, acetylation and SUMOylation. Here we highlight several important modification sites. Eight ISGylation sites have been reported, ① among which K41 is the main site of ISGylation, which disrupts the Interaction of the NS1 RBD with Importin-α. ②T49 position modification impairs the binding ability of NS1 to RIG-I, dsRNA and TRIM25 ternary complexes. ③ Phosphorylation modification of S205 enhances the binding ability to DDX21 and enhances polymerase activity. ④ Acetylation modification of this site possesses the ability to bind to CPSF30.

Post-translational modifications (PTM) have significant effects on the localization of the NS1 protein, its ability to interact with other proteins, and its study will help to better understand the unique biological functions of the NS1 protein and its impact on the pathogenic ability of influenza viruses (**Figure 1**).

#### Phosphorylation

Phosphorylation is a basic molecular switch, which can change the function of protein and regulate the biological process of protein. The reported phosphorylation sites are S42, S48, T49, T197, S205 and T215. T215 may be phosphorylated by cyclin-dependent kinases (CDKs) or extracellular signal-regulated kinases (ERKs) (Hale et al., 2009; Matsuda et al., 2010). The phosphorylation of T215 may affect the subcellular localization of NS1: because it is the NLS2 region, it may lead to the interruption of nuclear localization of NS1 (Melén et al., 2007). And in different influenza subtypes, the mutation of amino acids has different effects on phosphorylation (Hsiang et al., 2012). T49 was identified as a phosphorylation site (Kathum et al., 2016). In this study, the dynamics of NS1 phosphorylation in virus life cycle were studied for the first time. The results showed that T49 of NS1 was only phosphorylated in the later stage of replication cycle. T49 is located in the RBD area of NS1, and phosphorylation of this site reduces its binding ability of dsRNA, TRIM25 and RIG-I complex. Therefore, it can be concluded that the inhibition of NS1-dependent interferon activity is closed by phosphorylation at T49 in the later stage of the replication cycle. S205 is a newly discovered phosphorylation site, which is also one of six amino acid changes in NS1 of pandemic H1N1 viruses. It can promote NS1-DDX21 binding and enhance viral polymerase activity (Patil et al., 2021).

#### SUMOylation

SUMOylation of NS1 protein K219 and K221 of H5N1 subtype avian influenza virus increased the stability of protein and promoted the virus replication of highly pathogenic H5N1 strain (Xu et al., 2011). Meanwhile, PIAS2 protein from duck can promote the SUMOylation of NP through SUMO1, and then promote the replication of H5N1 AIV (Zu et al., 2020). NS1 SUMOylation at K131 is associated with the rapid replication of H1N1 IAV (Way et al., 2020).

However, SUMOylation of NS1 protein can also inversely regulate or antagonize the innate immunity of the host. SUMOylated NS1 protein can make RNAPII penetrate into the downstream foreign genes and nearby genes, and enhance the inhibition of host transcription reaction by interfering with the termination of RNAPII (Zhao et al., 2018).

In addition, when the virus invades the host, it usually leads to the decrease of SUMOylated TRIM28, which leads to the decrease of its ability to repress transcription. It also makes the interferon defense system activated by dsRNA which depends on RIG-1, MAVS, TBK1 and JAK1. Interestingly, the expression of interferon-stimulated genes is still limited, because influenza A virus NS1 greatly limits the production of many ISGs. Of course, in this process, TRIM28 triggered by influenza lost its SUMOylation, which reduced its inhibition on endogenous retrovirus (ERV). ERVs is up-regulated during influenza virus infection and stimulates antiviral immunity to help defend against new invasive pathogens (Schmidt et al., 2019).

#### ISGylation

Interferon-stimulated gene 15(ISG15) is an interferon-stimulated gene consisting of two ubiquitin-like (UBL) structural domains (Narasimhan et al., 2005). ISG15 is covalently linked to the target protein *via* the carboxy-terminal LRLRGG motif in a process called ISGylation (Potter et al., 1999). ISGylation requires a three-step enzymatic cascade reaction consisting of an E1 activating enzyme, an E2 binding enzyme, and an E3 ligase. During the initial phase of the innate response, ISG15 is a highly upregulated interferon-stimulated gene protein (ISGs) (Freitas et al., 2020). ISGylation of viral proteins can interfere with their localization, protease activity; disrupt their interaction with host proteins or other viral proteins; disrupt their own oligomerization and geometry; or affect other functions, ultimately leading to reduced viral replication or altered host immune status. The specific mechanism of its interaction with NS1 protein will be described in the “the interaction between NS1 and ISGs” section.

#### Acetylation

Acetylation is an important post-translational modification. Previous studies have found that the acetylation of K77, K113 and K229 of NP protein is very important for virus polymerase activity and replication (Giese et al., 2017). The K108R deacetylation mutation in NS1 protein decreased the replication and virulence of WSN-H1N1 virus, while the constant acetylation mimics mutant virus weigh 108Q showed similar replication and pathogenicity to wild type virus (Ma et al., 2020). Besides, K108R mutation significantly reduced the IFN-β antagonistic activity of NS1 protein, which may be due to the disruption of gene expression in inefficient common host genes, while R108K restored the ability to block common host genes and bind to CPSF30 (Hale et al., 2010).

Post-translational modification is the process of adding modification groups or small-molecule proteins to specific amino acid residues on a protein and thus covalently processing the modification, which is a new direction in the study of host-virus interaction mechanisms. Here we summarize four typical post-translational modifications that occur on the influenza virus NS1 protein. This is a new perspective that leads us to examine the unique role of NS1 proteins in the antiviral response. Overall, post-translational modifications are a major means of host restriction of viral replication, but as viruses have mutated, some modifications have become strain-specific. ISGylation is a typical means of host restriction of viruses that effectively restricts NS1 protein dimerization, but H5N1 strains have fewer ISGylation sites compared to H1N1 subtype, which may which may explain why this subtype has become highly pathogenic influenza (Tang et al., 2010), all of which suggest that NS1 undergoes adaptive mutations in a way to increase the reproduction capability of the virus by breaking through the limitations of innate immunity; acetylation of NS1 protein is a novel modification that has not been studied more thoroughly, but with the development of various omics methods, more types of modifications as well as more important modification sites should be uncovered in the future, which It is beneficial to discover new therapeutic targets and lay a good foundation for new antiviral drugs. However, post-translational modifications are also a double-edged sword, as NS1 can use SUMOylation to enhance its ability to inhibit host gene expression, which requires us to consider whether there is a potential balance when multiple modifications occur simultaneously, or which modifications play a dominant role under the action of NS1. This question may need to be explored using dynamic modifications or multi-omics crosstalk approaches.

## Four “Tricks” Played by NS1 Against Innate Immunity

**Figure 2:**
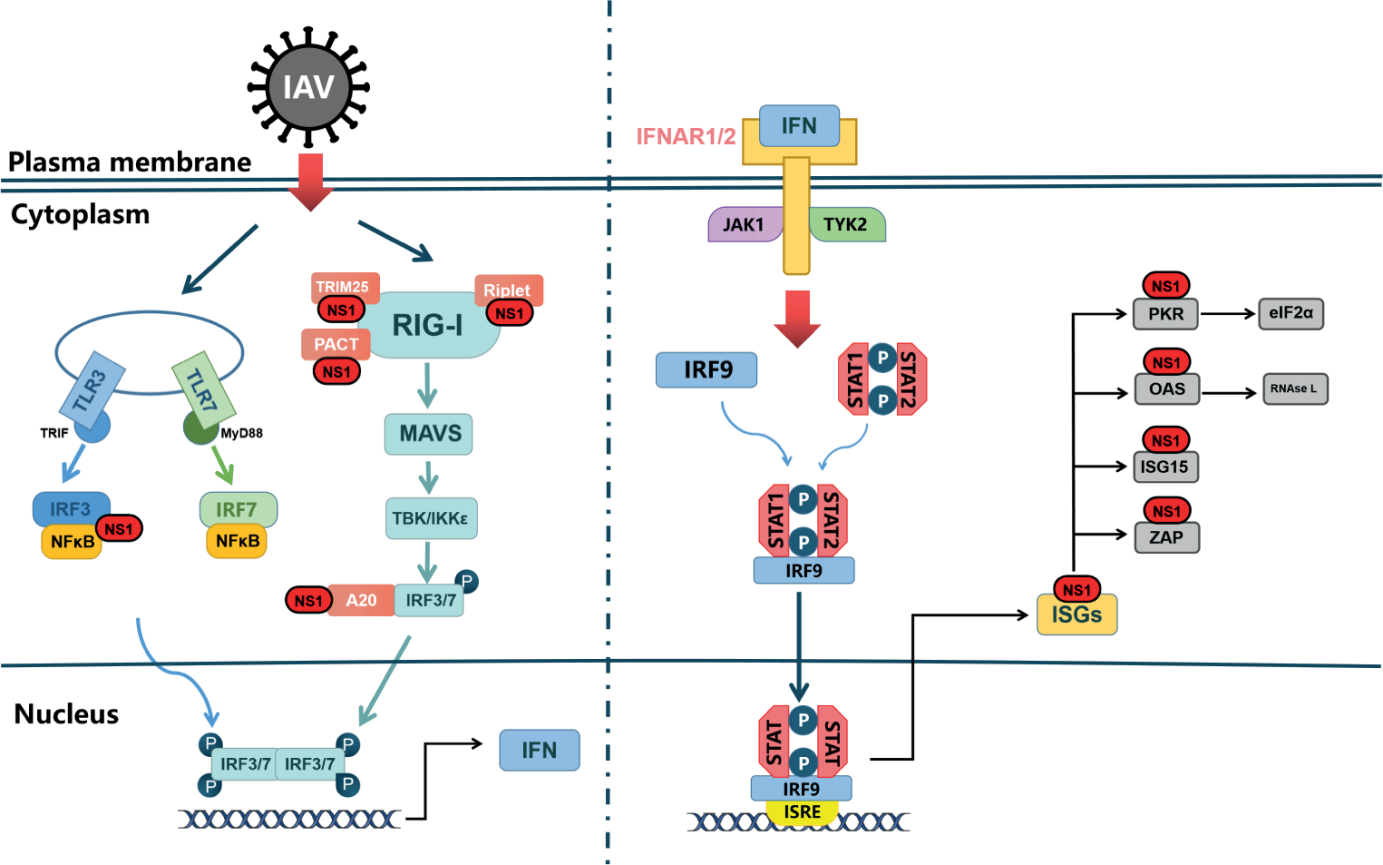
NS1 antagonistic to web of Interferon signal. TLRs and RLRs pathways are the main pathways in the type I interferon reaction induced by influenza A virus. TLR can signal through different junction proteins to trigger a signal transduction cascade leading to activation of IRF3/7 thereby inducing interferon production. Among them, TLR3 uses TRIF as a junction protein, while MyD88 is a junction protein of TLR7. RIG-I binds to viral RNA and signals down through MAVS, and activation of IRF3/7 and NF-κB factors induces synthesis of type I interferon mRNAs. IFNAR1/2, through JAK1 and TYK2, causes STAT1, STAT2 phosphorylated into dimers, which together with IRF-9 form ISGF3 that translocates to the nucleus, stimulating the expression of ISGs. In virus-infected cells, NS1 can interact with various host proteins to suppress cellular mRNAs through RIG-I receptor interactions as well as competitive binding of dsRNA. NS1 can also interact with various ISGs to antagonize the host’s natural immune response.

IAV has evolved different strategies to maximize inhibition of IFN expression. In particular, the viral NS1 protein antagonizes the host interferon response through several different molecular mechanisms (Krug, 2015). **Figure 2** shows the signal transduction pathway of type I interferon and the countermeasures of influenza virus NS1 to limit interferon induction. Generally speaking, IAV inhibits the antiviral immune response of the host, mainly by inhibiting the production of IFNs and the antiviral activity of ISGs, while the NS1 of the virus is mainly responsible for antagonizing the antiviral response of IFN.

Many studies have demonstrated the important role of NS1 protein in evading the antiviral mechanism of interferon by using recombinant influenza virus with full or partial deletion of NS1 (García-Sastre et al., 1998; Talon et al., 2000). In IFN-intact cells, the replication ability of IAV without the NS1 gene was much lower than that of wild type virus. On the contrary, in IFN-deficient Vero cells, the replication ability of the NS1 deficient virus was almost the same as that of wild type virus (García-Sastre et al., 1998).

Other researchers truncated the C-terminal of NS1 protein and found that in cells with intact IFN system, the recombinant virus encoding only NS1 1-124 amino acid had little effect on replication, but the expression of truncated NS1 protein was very low, while mutations R38 and K41 significantly affected the ability of NS1 to inhibit IFN production (Pichlmair et al., 2006). In addition, cells infected with NS1 deficient viruses could induce more IFN- α and IFN- β than wild type viruses (Fernandez-Sesma et al., 2006). A recent study found that the conserved residue Y84 in the SH2-binding domain of NS1 is essential for limiting the response of interferon. Y84F mutation offsets IFN-induced STAT phosphorylation and downregulation of IFNAR1 expression (Wang et al., 2017).

These experiments show that NS1 is an important factor for influenza virus to inhibit host innate immune response. Generally speaking, NS1 can inhibit the antiviral system against the host through three mechanisms: first, directly inhibit the production of IFN, including interaction with RIG-I receptors and competitive binding to dsRNA; second, inhibit the expression of mRNAs; third, the interaction with ISGs; in addition, NS1 can also promote viral replication by regulating apoptosis, which has received increasing attention in recent years.

### Inhibiting Interferon Production

#### NS1 Suppresses the Signal Pathway of RIG-I

After the influenza virus invades the host, the RIG-I-mediated antiviral signaling pathway can effectively induce the expression of interferon, which in turn mediates a series of antiviral responses (Hu and Shu, 2018). However, the signaling process is tightly regulated by post-translational modifications of RIG-I, and TRIM25 and RIPLET are important ubiquitin ligases in promoting RIG-I signaling activation (Oshiumi et al., 2013).

Previous studies found that NS1 inhibited the activation of IRF3 (Talon et al., 2000), NF-κB (Wang et al., 2000). Subsequent studies have shown that this inhibition occurs at the starting point of the RIG-I signal pathway: the RIG-I receptor.NS1 can interact with two kinds of E3 ubiquitin ligase: TRIM25 (Gack et al., 2009) and Riplet (Rajsbaum et al., 2012). TRIM25 strips the RNA template of viral polymerase and inhibits the extension of RNA chain by restricting the movement of RNA in the polymerase complex (Meyerson et al., 2017).

NS1 can inhibit the oligomerization of TRIM25 itself, and then inhibit the activation of RIG-I. On the other hand, the E96A/E97A mutation of NS1 loses the ability to bind TRIM25, weakens the replication of the virus and induces the expression of a high level of IFN (Gack et al., 2009). NS1 can also bind to the Riplet, inhibiting the Riplet catalyzes the RIG-I activation, and unlike TRIM25, the E96/E97 residue of NS1 does not participate in inhibiting the Riplet (Rajsbaum et al., 2012). Riplet is a condition to the RIG-I signal for TRIM25: Riplet-mediated polyubiquitination of the K63 released RIG-I RD autorepression, permitting access to the RIG-I protein for positive factors (Oshiumi et al., 2013).

NS1 has been shown to interact directly with the second caspase activation and recruitment domain(CARD2) of RIG-I, which depends on RBD of NS1 (Jureka et al., 2015). A recent study revealed the underlying mechanism of this phenomenon, in which the authors identified a CCAAT enhancer-binding protein (C/EBPβ) binding site in the RIG-I promoter as a repressor. NS1 promotes C/EBPβ phosphorylation and recruitment to the RIG-I promoter as a C/EBPβ-NS1 complex, which has a negative regulatory effect on RIG-I (Kumari et al., 2020). The study of strain-specific polymorphisms also revealed that the NS1 R21Q mutation resulted in a significant upregulation of RIG-I signaling. The R21Q mutation in the NS1 RBD significantly disrupts its ability to interact with the CARD and downregulates the ability to compete with TRIM25 for binding RIG-I, resulting in enhanced activation levels of the IFN-β promoter (Jureka et al., 2020).

#### NS1 Binds Cellular RNA and DNA to Block Transcription of Antiviral Genes

The dsRNA binding characteristics of the RBD region of NS1 protein play an important role in its inhibition of IFN pathway. The traditional view is that the competition between RBD and RIG-I for PAMPs, reduces the recognition of PAMPs by RIG-I, but it is found that the affinity between RBD and dsRNA is very low (Chien et al., 2004), which indicates that NS1 is unlikely to compete with RIG-I to combine dsRNA. However, the RNA binding properties of NS1 proteins may help NS1 bind to host proteins with RNA binding ability to form complexes, thereby inhibiting the production of IFN, such as RIG-I, dsRNA and PKR (Onomoto et al., 2012).

In addition to dsRNA, NS1 can bind to synthetic dsDNA in a sequence non-specific manner. This interaction inhibits the initiation of the transcriptional mechanism on the synthesis of DNA, thus preventing the transcriptional response *in vitro*. In infected cells, NS1 inhibits the recruitment of Pol II to IFNB1 exons and promoters. Therefore, NS1 protein can bind to the DNA of transcriptional active genes and attenuate their expression. This may potentially lead to a decrease in the expression of IFN and ISGs, resulting in impaired antiviral response of infected cells (Anastasina et al., 2016).

#### Indirect Regulation of the RIG-I Signaling Pathway by NS1 Protein

NS1 protein can also indirectly affect RIG-I signal pathway by interacting with host factors. NS1 indirectly regulates RIG-I signal by inducing ubiquitin-editing protein A20 during infection. A20 inhibits IRF3-mediated induction of type I IFN and ISGs (Feng et al., 2017).

IAV NS1 protein can induce host cells to express MCPIP1, which can negatively regulate cellular inflammatory response. MCPIP1 degrades RIG-I mRNA through its RNase activity, then inhibits the phosphorylation of IRF3, the expression of IFN and ISG genes, and finally promotes the replication and proliferation of IAV (Sun et al., 2018).

### Suppressing Host Gene Expression

In addition to targeting important proteins in the interferon pathway, IAV NS1 proteins can also block the expression of general genes.NS1 binds to CPSF30, blocking the binding of the whole CPSF complex to pre-mRNA, and finally inhibiting the cleavage and polyadenylation of mRNA (Nemeroff et al., 1998; Twu et al., 2006). The four subunits of influenza virus polymerase PB1, PB2, PA and NP could stabilize the binding of NS1-CPSF (Twu et al., 2007). The binding of NS1 to CPSF30 also inhibited the nuclear-cytoplasmic transport and splicing processing of host mRNA (Kuo and Krug, 2009).

The key amino acid site for the formation of the NS1-CPSF30 complex has been identified, such as E55/E66/F133(Amino acid co-mutation) (Li et al., 2018), I64T (DeDiego et al., 2016), F103L/M106I (Dankar et al., 2011; Dankar et al., 2013; Ayllon et al., 2014; Rodriguez et al., 2018), K186E (Nogales et al., 2017; Chauché et al., 2018) and so on. The mutation of F103L and M106I altered the binding ability of NS1 protein to CPSF of various subtypes of influenza viruses, including H3N2 (Dankar et al., 2011), H5N1 (Dankar et al., 2013), H7N9 (Ayllon et al., 2014), and H9N2 (Rodriguez et al., 2018), and improved their ability to antagonize interferon, resulting in a certain degree of enhancement of virulence *in vivo* and *in vitro*.

NS1 forms an inhibitory complex with NXF1/TAP, p15/NXT, Rae1/mrnp41, and E1B-AP5, which are key constituents of the mRNA export machinery that interact with both mRNAs and nucleoproteins,to prevent the output of mature mRNA (Satterly et al., 2007). Also, NS1 can bind poly A chain, which prevents mature mRNA from being exported from the nucleus (Qian et al., 1994; Qiu and Krug, 1994).

### Interacting With ISGs

Interferon-stimulating genes exert their antiviral functions through different mechanisms, including direct targeting of viruses, such as interferon-stimulating genes that can degrade viral RNA, inhibit translation, block the release of virions, and regulate the activity of host responses, including interferon response, NF-κB signal, cell hole death and other active responses. NS1 protein can not only inhibit the production of IFN, but also directly antagonize ISGs.

#### Protein Kinases R

PKR, a RNA-binding protein kinase, is expressed in an inactive form in mammalian cells and is significantly up-regulated after interferon treatment (Hovanessian, 1989). PKR can be activated by dsRNA (Hatada et al., 1999). It can also be activated by the PACT protein in the absence of RNA. Activated PKR inhibits translation mainly by phosphorylating eukaryotic translation initiation factor 2a (elF2a), which restricts the synthesis of all proteins, including viral proteins, in infected cells (García et al., 2006; García et al., 2007).

However, in influenza virus infected cells, PKR could not be activated, because NS1 RBD 123-127 residues could bind to PKR to counteract the effect of PKR (Min et al., 2007). The interaction between NS1 and PKR junction region (Li et al., 2006) prevents PKR from being activated due to conformational changes caused by recognition of dsRNA or binding to PACT. This mechanism enables NS1 to escape the inhibition of dsRNA and PACT-mediated translation through PKR.

In addition, cells infected with influenza virus can activate p58IPK protein, which is a cellular PKR inhibitor that is activated during IAV infection and negatively regulates PKR (Goodman et al., 2007).

#### OAS-RNase L

OAS is an IFN-induced antiviral protein that is also activated by dsRNA and further binds and activates ribonuclease L (RNase L), which hydrolyzes viral or cellular cellular single-stranded RNA, preventing viral replication (Silverman, 2007). These degraded RNA products may be recognized by RIG-I-like receptors and activate the production of IFN. Studies have shown that NS1 protein inhibits the activation of OAS by competing with OAS to bind dsRNA, through the dsRNA binding characteristics of RBD. The R38A mutation in the NS1 protein of H3N2 virus reduced replication1000-fold, but viral replication was significantly enhanced after silencing or knockdown of RNase L, suggesting that OAS-RNase L can inhibit the replication of influenza virus (Min and Krug, 2006; Min et al., 2007).

#### Interferon Stimulating Gene 15

ISG15 is a broad-spectrum antiviral protein that acts by modifying many viral proteins, such as: Coxsackie virus B3 (Rahnefeld et al., 2014), dengue virus (Hishiki et al., 2014), and pseudorabies virus (Liu et al., 2018).

ISGylation of influenza NS1 protein is the first reported viral protein (Zhao et al., 2010). NS1 is essential for viral replication because it inhibits the production of type I interferon (Wang et al., 2000). The ISGylation of influenza A virus NS1 prevents it from forming homodimer, in which the modification of lysine at position 41 (K41) destroys the interaction between NS1 and Importin-α (Zhao et al., 2010), thus inhibits the nuclear translocation of NS1 and makes the virus vulnerable to interferon. ISGylation of NS1 at different sites also disrupts its interactions with RNA targets, such as U6snRNA, dsRNA, which limits the ability of NS1 to destroy the innate immune response and interfere with the host antiviral response. Consistent with this, IAV exhibited enhanced replication capacity in ISG15 knockout A549 cells (Tang et al., 2010).

It is worth noting that different subtypes of influenza viruses and different types of cells have different effects on the screening results, so it is necessary to study the interaction between specific strains and ISG15 according to the seasonal epidemic. In addition, the selection of different hosts also has a great impact on the results of the study, for example, the homology of ISG15, among species is very low. In addition, the preliminary studies of many experiments are carried out *in vitro*, and the use of cells related to *in vitro* experiments and the level of host proteins expressed in different cell types should also be considered.

#### Zinc Finger Antiviral Protein

Zinc finger antiviral protein (ZAP) is an antiviral protein in mammals, which can effectively inhibit the replication of many kinds of RNA viruses (such as retroviruses, alphaviruses, linear viruses, etc.) (Müller et al., 2007). Two kinds of proteins with different sizes can be obtained from the mRNA of human ZAP proteins by different splicing methods, and the higher molecular weight is hZAP-L, and the smaller is hZAP-S. IFNs or IPS-1 treatment can up-regulate the expression of ZAP protein, while ZAP protein can recognize and bind the ZAP response element (ZAP-responsive elements, ZRE) on viral RNA, and then recruit a variety of exonucleases to degrade viral RNA. The ZAP protein has been shown to use the zinc finger structural domain to recognize the viral ZRE and thus degrade the hepatitis B virus (HBV) precursor RNA to inhibit HBV replication (Mao et al., 2013). Tang et al. found that the antiviral activity of ZAPS was antagonized by viral protein NS1. This is because NS1 can inhibit the binding of ZAPS to target mRNA and decrease its activity. These results reveal the different mechanisms of the interaction between ISGs and IAV NS1 proteins (Tang et al., 2017).

### Regulating Apoptosis

Apoptosis is a programmed cell death process which is involved in a variety of important biological processes, including resistance to viruses. The specific mechanisms by which IAV regulates apoptosis are complex and multifaceted, as it encodes several proteins that regulate this process, such as NA, PB1-F2, M1, M2 and NS1. Here we focus on NS1 protein. It is unique compared to other proteins in that it not only inhibits apoptosis, but also promotes it.

Preliminary studies found that the PR8 virus NS1 protein may inhibit host cell apoptosis through a type I IFN-dependent mechanism (Zhirnov et al., 2002). Expression of NS1 singly inhibits the transcriptional activity of p53 and apoptosis (Wang et al., 2010). In addition, it has been found that NS1 protein initiates anti-apoptotic PI3k-Akt signaling in the early and middle stages of infection to protect cells from rapid apoptotic death (Zhirnov and Klenk, 2007; Ehrhardt et al., 2007). However, another study found that deletion of the functionally intact NS1 protein promotes apoptosis, but this is not due to the inability to activate PI3K (Jackson et al., 2010). A study also found that NS1 of an H3N2 influenza virus strain antagonized MAVS-mediated intrinsic apoptosis, which was significantly upregulated at both the transcriptional and protein levels early in infection. The H9N2 subtype strain differs from other strains in that its NS1 inhibits extrinsic apoptosis by inhibiting the induction of FasL (Xing et al., 2009).

Georgia et al. suggested that since viral replication must be completed before cells are disintegrated by apoptosis, expression of anti-apoptotic viral proteins could promote viral multiplication prior to cell death (Atkin-Smith et al., 2018). Based on the current study, it also appears that the NS1 protein exerts its anti-apoptotic properties mostly at the early stage of infection. At different stages of the influenza virus replication cycle, the balance between promotion and inhibition of apoptosis is in different states. It has been suggested that dsRNA can act as a pro-apoptotic factor in the early stage of infection, but the presence of NS1 prevents the activation of NF-κb and the synthesis of IFN-β (Zhirnov et al., 2002), and the activation of PKR (Schultz-Cherry et al., 2001) is also blocked, so it presents a state of inhibition of apoptosis.

NS1 of influenza A virus can also induce apoptosis in MDCK and HeLa cells, and in the past decade or so, successive reports have revealed that NS1 can induce intrinsic and extrinsic apoptosis on different cellular cells (**Table 2**). NS1 exhibits a complex relationship with apoptosis, which is dependent on a variety of factors, such as influenza virus subtype and cell type. The NS1 protein encoded by H5N1 has a rapid-inducing ability to induce apoptosis compared to all other subtypes (Lam et al., 2011). This may be due to the fact that NS1 of the H5N1 isoform can downregulate HSP90, which may play an important role in the induction of apoptosis (Mukherjee et al., 2012). It has been suggested that influenza virus-induced apoptosis of lymphocytes may be an important pathogenic mechanism of highly pathogenic influenza viruses (Schultz-Cherry et al., 2001), suggesting an important connection between apoptosis and pathogenicity of influenza viruses. Infection with the H5N1 subtype, a highly pathogenic influenza virus, is accompanied by the rapid development of primary pneumonia (To et al., 2001; de Jong et al., 2006), which has also been shown to be somehow associated with apoptosis (Tumpey et al., 2000; To et al., 2001).However, it has also been found that the temporal distribution of apoptosis induced by different subtypes of influenza is similar. This may be due to its use of transfection, which allows for approximate synthesis of NS1 in different subtypes, whereas previous studies have infected intact viral particles (Lam et al., 2011).

**Table 2:** Different subtypes of IAV NS1 proteins induce apoptosis in various cell types.

IAV	Cell line	Mechanism of apoptosis induction	References
A/Chicken/shaanxi/01/2011 (H9N2)	COEC (chicken oviduct epithelial cells)	Activating ROS accumulation and mitochondria-mediated apoptotic signaling induces apoptosis in COECs	(Qi et al., 2016)
A/chicken/Zhejiang/DTID-ZJU01/2013(H7N9)	A549	Increasing the phosphorylation of p53 and inducing mitochondrial impairment leading to the accumulation of p53	(Yan et al., 2016)
A/Beijing/501/2009 (H1N1)	A549	Interacting with cellular β-tubulin, disrupting normal cell division and inducing apoptosis	(Han et al., 2012)
A/Aquatic bird/India/NIV-17095/2007(H11N1)	293T	Down-regulation of HSP90 expression to induce apoptosis	(Mukherjee et al., 2012)
A/chicken/Jilin/2003 virus (H5N1)	Human alveolar basal epithelial cells	Inducing apoptosis via the caspase-dependent pathway	(Zhang et al., 2010)
A/chicken/Jilin/2003 (H5N1) A/swine/Colorado/1/1977 (H3N2)	A549	Competing with apaf-1 for binding hsp90 leads to apaf-1 oligomerization, which in turn promotes apaf-1 interaction with cytc, leading to activation of caspase 9 and caspase 3, leading to apoptosis.	(Zhang et al., 2011)
A/HongKong/483/97 (H5N1)	Human airway epithelial cells (NCI-H292 cells)	Activating the death receptor signaling cascades, and triggering the caspase cascade, resulting in apoptosis	(Lam et al., 2008)
A/Puerto Rico/8/34 (H1N1)	Primary human macrophages	Controlling caspase-1 activation, thus repressing the maturation of pro-IL1b-, pro-IL18- and caspase-1-dependent apoptosis	(Stasakova et al., 2005)
A/Turkey/Ontario/7732/66 (H5N9)	MDCK/HeLa	Triggering the caspase cascade, resulting in apoptosis	(Schultz-Cherry et al., 2001)

In conclusion, NS1 has both apoptosis-promoting and apoptosis-inhibiting functions, which will be complicated for understanding the role of apoptosis in the pathogenesis of influenza viruses and may be related to different virus subtypes and cell types. However, studies on apoptosis will better contribute to our understanding of viral pathogenesis, especially the different pathogenic mechanisms of highly pathogenic and low pathogenic influenza viruses and facilitate the development of new therapies against influenza virus infections.

## Concluding Remarks

The interaction between virus and host is a complex and dynamic network, and its influencing factors are widely involved, especially because influenza A virus mutates more frequently and its NS1 is prone to adaptive mutations. Under such conditions, we should focus on the seemingly simple process of “virus invading host” from the perspective of connection and development. In recent years, with the development of technologies such as transcriptome, proteomics and metabolomics, we can examine the relationship between them from all levels. This also makes the exploration of the pathogenic mechanism of influenza virus more and more in-depth.

As a key protein in the “game” between influenza virus and host, NS1 can antagonize the activation of interferon system, the most powerful antiviral response of the host, from many levels, which also highlights its important position. As summarized in this paper, NS1 protein inhibits the dsRNA-mediated host antiviral pathway, or directly inhibit the production of IFN by targeting RIG-I; at the same time, it can regulate the production of mRNA and indirectly inhibit interferon signal pathway. NS1 can also interact with ISGs to antagonize the antiviral response of the host and with the help of quantitative proteomics, more ISGs interacting with influenza virus has been found (Hu et al., 2017).

In addition, NS1 affects apoptosis in different ways, but exactly which pathway or which protein is in the dominant position is not clear, which is a valuable research direction. The game between organism and virus is destined to be a protracted war, the host has also evolved various mechanisms to limit influenza viruses, and post-translational modifications are an area of great interest. In recent years, with the development of Bioinformatics technology and omics technology, new modifications such as crotonylation (Yu et al., 2020) and lactylation (Zhang et al., 2019) have been reported one after another. The regulatory effect of PTM on influenza virus has a broad research prospect.

Going forward, research on NS1 proteins will have to link viral and host factors, which may, of course, become more complex. Understanding the “virus-host interaction interface” by identifying the potential mechanisms by which different restriction factors inhibit influenza viruses is critical, and this will also help to identify and evaluate their potential as new antiviral therapeutic targets.

## Author Contributions

Z-xJ originally drafted the manuscript and edited the manuscript. X-qW and X-fL revised the manuscript. All authors contributed to the article and approved the submitted version.

## Funding

This work was supported by the National Natural Science Foundation of China: 31772755, 32072892, 32072832; by the National Key Research and Development Project of China: 2016YFD0500202–1; by the Earmarked Fund For China Agriculture Research System: CARS-40 and by the Priority Academic Program Development of Jiangsu Higher Education Institutions (PAPD).

## Conflict of Interest

The authors declare that the research was conducted in the absence of any commercial or financial relationships that could be construed as a potential conflict of interest.

## Abbreviations

5’ ppp-dsRNA, 5’ triphosphate double stranded RNA; CARD, Caspase activation and recruitment domain; C/EBPβ, CCAAT enhancer-binding protein; CDKs, Cyclin-dependent kinases; DCs, Dendritic cells; ED, Effector domain; ERV, Endogenous retrovirus; elF2a, Eukaryotic translation initiation factor 2a; ERKs, Extracellular signal-regulated kinases; IRF9, IFN regulatory factor 9; ISRE, IFN-stimulatory response element; IAV, Influenza A virus; IRF3/7, Interferon regulatory Factor 3/7; IFN, Interferon; ISG15, Interferon-stimulated gene 15; IFNAR, Interferon-α/β receptor; LR, Linker region; MyD88, Myeloid Differentiation Factor 88; NS1, Non-structural protein 1; NLS, Nuclear localization signal; PAP, Poly A polymerase; PAMPs, Pathogen-associated molecular patterns; PRs, Pattern recognition receptors; CPSF30, Cleavage and polyadenylationspecificitv factor 30 kDa; PTM, Post-translational modification; RLRs, Retinoic acid-inducible gene I-like receptors; RNase L, Ribonuclease L; RNP, Ribonucleoprotein; RBD, RNA binding domain; CTT, Terminal tail; TLRs, Toll-like receptors; ISGF3, Transcriptionally activate IFN-stimulated gene factor 3; UBL, Ubiquitin-like; ZAP, Zinc finger antiviral protein.
